# Curcumin Protects against UVB-Induced Skin Cancers in SKH-1 Hairless Mouse: Analysis of Early Molecular Markers in Carcinogenesis

**DOI:** 10.1155/2012/593952

**Published:** 2012-07-19

**Authors:** Kuen-Daw Tsai, Jung-Chung Lin, Shu-mei Yang, Min-Jen Tseng, Jeng-Dong Hsu, Yi-Ju Lee, Jaw-Ming Cherng

**Affiliations:** ^1^Department of Internal Medicine, China Medical University Beigang Hospital, Yunlin 651, Taiwan; ^2^School of Chinese Medicine, College of Chinese Medicine, China Medical University, Taichung 404, Taiwan; ^3^Institute of Molecular Biology, Department of Life Science, National Chung Cheng University, Chiayi 621, Taiwan; ^4^Cellular Virology Unit, Division of Viral Hepatitis, Centers for Disease Control and Prevention, Atlanta, GA 30333, USA; ^5^Department of Pathology, Chung Shan Medical University Hospital and Chung Shan Medical University, 110, Jianguo N. Road, Sec. 1, Taichung 402, Taiwan; ^6^Department of Internal Medicine, Chung Shan Medical University Hospital and Chung Shan Medical University, 110, Jianguo N. Road, Sec. 1, Taichung 402, Taiwan

## Abstract

Curcumin (CUR) has been shown to possess a preventive effect against various cancers and interfere with multiple-cell signaling pathways. We evaluated the protective effects of CUR in regression of UVB-induced skin tumor formation in SKH-1 hairless mice and its underlying early molecular biomarkers associated with carcinogenesis. Mice irradiated with UVB at 180 mJ/cm^2^ twice per week elicited 100% tumor incidence at 20 weeks. Topical application of CUR prior to UVB irradiation caused delay in tumor appearance, multiplicity, and size. Topical application of CUR prior to and immediately after a single UVB irradiation (180 mJ/cm^2^) resulted in a significant decrease in UVB-induced thymine dimer-positive cells, expression of proliferative cell nuclear antigen (PCNA), terminal deoxynucleotidyl transferase-mediated dUTP nick end labeling, and apoptotic sunburn cells together with an increase in p53 and p21/Cip1-positive cell population in epidermis. Simultaneously, CUR also significantly inhibited NF-**κ**B, cyclooxygenase-2 (COX-2), prostaglandin E2 (PGE2), and nitric oxide (NO) levels. The results suggest that the protective effect of CUR against photocarcinogenesis is accompanied by downregulation of cell proliferative controls, involving thymine dimer, PCNA, apoptosis, transcription factors NF-**κ**B, and of inflammatory responses involving COX-2, PGE2, and NO, while upregulation of p53 and p21/Cip1 to prevent DNA damage and facilitate DNA repair.

## 1. Introduction 

Ultraviolet (UV) light has been well documented as a complete carcinogen responsible for initiation and promotion of both basal and squamous cell carcinomas. Long-term exposure of the skin to UV radiation results in degenerative processes involved in photoaging and photocarcinogenesis [1]. Furthermore, exposure to UV accounts for approximately 65% of melanoma and 90% of basal and squamous cell carcinoma [2, 3]. Direct absorption of UV by DNA leads to the formation of cyclobutane pyrimidine dimers and pyrimidine-pyrimidone dimers of DNA bases [4, 5]. In addition, UV-induced reactive oxygen intermediates can also cause DNA adducts and other types of oxidative damage [1]. Like many chemical tumor promoters, UV also elicits inflammation, epidermal hyperplasia, and changes in the expression of numerous genes associated with proliferation and differentiation, eicosanoid and cytokine production, and growth factor synthesis and responsiveness [1]. Thus, UV is considered a complete carcinogen because it can initiate and induce cancer growth in the absence of any other carcinogen [6]. This diversity of responses suggests that there are probably multiple processes that could be effective targets for prevention.

Curcumin (CUR, diferuloylmethane) is a major component of turmeric, a yellow spice derived from dried rhizomes of *Curcuma longa*. CUR has been found to have antioxidant, anti-inflammatory, and antitumor activity in a variety of animal models of human diseases [7–10]. CUR has already entered clinical trials because of its potent anti-inflammatory, anticarcinogenic, and free radical scavenger properties [11]. The mechanisms by which CUR affects multiple biochemical and inflammatory conditions appear to be cell- and stimulus-specific and to involve effects on the cell's transcriptional machinery such as NF-*κ*B, COX-2 [9, 10], and redox homeostasis. CUR has direct antioxidant activities [8] and it is a potent inhibitor of prostaglandin synthesis [12]. Several clinical studies indicated that CUR exerted an anti-inflammatory activity was due in part to the inhibition of inducible isoforms of nitric oxide synthase (iNOS) [13] and COX-2 enzymes [13, 14]. Nitric oxide (NO) has been proposed to be important mediator of tumor growth [15] and overexpressed NOS has also been detected in several human tumors [16, 17]. In particular, the inhibition of COX-2 was significant in colon cancer cells, which makes CUR important as a colon cancer preventive agent [18]. The inhibition of the COX-2 enzyme is achieved by suppressing the activation of NF-*κ*B, a eukaryotic transcription factor [19]. 

We assessed the protective effects of CUR against photocarcinogenesis in the SKH-1 hairless mouse skin model. We found that CUR is a potent inhibitor against UVB-induced skin tumors. We further investigated its effect on early biomarkers associated with photocarcinogenesis. The findings and biological significance were reported in the present study. 

## 2. Materials and Methods

### 2.1. Animals, UV Light Source, and Chemicals

Inbred female SKH-1 hairless mice (5 weeks old) were purchased from Charles River Laboratories (Wilmington, MA, USA) and maintained in accordance with relevant guidelines and regulations for the care and use of laboratory animals of China Medical University. 

The UVB light source consisted of four FS40T12/UVB sunlamps (Philips, Amsterdam, The Netherlands), which emitted ~80% radiation in the range of 280 to 340 nm with a peak emission at 314 nm as monitored with SED240 photodetector with SPS300 filter and T input optic connected to an ILT1700 Research Radiometer. (International Light Technologies, Newburyport, MA, USA). The SPS300 filter removes wavelengths shorter than 280 nm and with the predominant emitting peak at 280–315 nm. The radiometer is calibrated on a regular basis against both a traceable standard lamp and against the laboratory radiation source. 

Mice were exposed to UVB irradiation for 2 min and 40 sec with a distance of 23 cm between the light source and the target skin. 

 Curcumin (diferuloylmethane) was purchased from Sigma-Aldrich, Inc. (St Louis, MO, USA) and was solubilized in acetone. 

### 2.2. Experimental Designs

**Figure 1:**
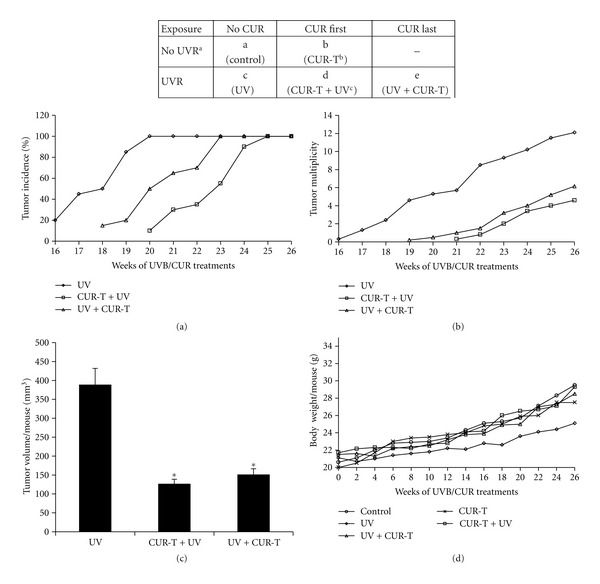
Experimental design and effect of CUR on UVB-induced skin photocarcinogenesis in SKH-1 hairless mouse. Upper panel, experimental design depicting variables and treatment groups both in long-term and short-term study. Mice were divided into 5 groups, a, b, c, d, and e. Twenty mice per group for long-term study and 5 mice per group for short-term study. (a) UVR, UVB irradiation (180 mJ/cm^2^), twice/week for long-term and single exposure for short-term study. (b) CUR-T, topical application of CUR, twice/week for 26 weeks (long-term study); once for short-term study. (c) CUR-T + UV, topical application of CUR 30 min prior to UVB; UV + CUR-T, topical application of CUR immediately after UVB irradiation. Dorsal skins were topically wet dressed with a filter paper soaked with 10 mmol CUR in 200 mL acetone. Lower panels, effect of CUR on UVB-induced skin photocarcinogenesis in SKH-1 hairless mouse. The results were obtained from the long-term regimen shown in the upper panel. Experiment was terminated at 26 weeks after UVB exposure. Percentage of tumor incidence (a), tumor multiplicity per mouse (b), tumor volume per mouse (c), and body weight per mouse (d) were recorded and analyzed. The data shown in (c) were mean ± SE (*bars*). In each case, the data shown were from 20 mice in each group. No tumors were observed in control and topically treated CUR alone groups. *P < 0.005 versus UVB group.

Both long-term and short-term studies were conducted to assess the effect of CUR on UVB-induced skin photocarcinogenesis in female SKH-1 hairless mice. The experimental design, variables, and treatment groups are depicted in Figure 1(a). The long-term regimen was designed to assess the effect of CUR on (a) percent tumor incidence, (b) tumor multiplicity (number of tumors per mouse), and (c) tumor volume per mouse, whereas the short-term study was for assessing early molecular biomarkers.

After UVB treatment, percent tumor incidence was documented by counting dorsal tumors and measuring their size at weekly intervals. Tumor size was measured using calipers, and tumor volume was estimated by the hemiellipsoid model formula: tumor volume = 1/2(4*π*/3) × (*l*/2) × (*w*/2) × *h*, where *l* = length, *w* = width, and *h* = height. 

Animals were killed at various time points, dorsal skin tumors were collected, fixed in 10% formalin for 8–10 h at 4°C, dehydrated in ethanol, cleared in xylene, and embedded in paraffin. Four *μ*m serial sections were cut and processed. Tumor formation was confirmed by histomorphologic analysis using hematoxylin and eosin (H&E) staining and various immunohistochemical analyses and examined by light microscopy.

### 2.3. Pathological Analysis of Sunburn and Apoptotic Cell Formation

Apoptotic cells are characterized by cell shrinkage and nuclear condensation, which attributes to their small, dense nuclei and eosinophilic cytoplasm that stain darker by H&E staining. Twenty-four hours after UVB exposure, the mice in were killed, and the dorsal skin was excised, fixed in 10% buffered formalin, and embedded in paraffin. Vertical sections of the skin (4 *μ*m thick) were cut and mounted on glass slides using H&E staining. Each section was examined under light microscopy for the formation of sunburn cells by two investigators in a blinded fashion. The microscopic examinations were performed by two investigators in a blind fashion. For every specimen, five to ten randomly selected fields were examined and counted at 400x magnification. Data were calculated as mean ± SE of 25 fields/5 mice/group. 

### 2.4. Immunohistochemical Analysis of Biomarkers

The apoptotic cells were detected by using the Dead End Colorimetric TUNEL system (Promega Corporation, Madison, WI, USA) according to the manufacturer's instructions. The apoptotic sunburn cells were stained conventionally with H&E and examined by light microscopy.

 To detect thymine dimer-positive cells, antithymine dimer antibody (Kamiya Biomedical Company, Seattle, WA, USA) was used. Endogenous peroxidase activity was blocked by incubation with 3% hydrogen peroxide for 10 minutes at room temperature. Slides were then incubated with 0.125% trypsin for 30 min at 37°C and then with 1 N HCl for 30 min at room temperature. The sections were then blocked with 5% goat serum for 10 min and incubated with peroxidase-conjugated monoclonal antithymine dimer antibody for 90 min at room temperature.

 For detection of p53, p21/Cip1,and PCNA, mouse monoclonal anti-p53 (LifeSpan BioSciences, Seattle, WA, USA), anti-p21/Cip1 (Acris Antibodies GmbH, Herford, Germany), or anti-PCNA (Biocare Medical, Concord, CA, USA) antibodies were used. After deparaffinization and re-hydration, skin sections were treated with 0.01 M sodium citrate buffer (pH 6.0) in a microwave for 5 min at full power for antigen-retrieval. Then, sections were quenched of endogenous peroxidase activity by incubating with 3% hydrogen peroxide for 5 minutes at room temperature. After that, sections were blocked with 5% rabbit serum for 30 minutes at room temperature and then incubated at room temperature for 2 h with anti-p53, anti-p21/Cip1, or anti-PCNA antibody for immunohistochemical analysis. 

Control sections were incubated with PBS only under identical conditions. After that, sections were detected using NovoLink Polymer Detection System (Novocastra Laboratories, Newcastle Upon Tyne, UK) according to the manufacturer's instructions. Sections were then visualized with 3,3′-diaminobenzidine (DAB), which reacts with peroxidase to give a brown reaction product. The sections were counterstained in hematoxylin, dehydrated and mounted. The microscopic examinations were performed by two investigators in a blind fashion. For every specimen, five to ten randomly selected fields were examined and counted at 400x magnification. Data were calculated as mean ± SE of 25 fields/5 mice/group.

### 2.5. Biochemical Analysis of NO, COX-2, and PGE2 Activities

The frozen skin specimens were pulverized in liquid nitrogen. The powder was suspended in cell lysis buffer (Sigma-Aldrich, Inc.,) supplemented with protease (Complete, Roche) and phosphatase (PhoStop, Roche) inhibitors and sonicated before centrifugation at 12,500 xg for 20 min. The supernatants were collected and used for quantitative analysis of NO (BioVision, Mountain View, CA, USA), COX-2 (USCN LIFE, Wuhan, China), and PGE2 (R&D System, Minneapolis, MN, USA), using ELISA kits following the manufacturers' protocols.

### 2.6. Analysis of NF-*κ*B DNA Binding Activity

For analyzing transcription factor NF-*κ*B binding activity to DNA, nuclear proteins were prepared as described previously [20] and quantified the binding activity using TF ELISA kit (Panomics, Fremont, CA, USA) following the manufacturer's protocol. This method is faster, easier, and more sensitive than electrophoretic mobility shift assays and does not require the use of radioactivity. 

### 2.7. Statistical Analysis

Data were presented as means ± standard error. The evaluation of statistical significance was determined by one-way analysis of variance (ANOVA) followed by Bonferroni *t*-test for multiple comparisons. A *P* value less than 0.05 was considered statistically significant.

## 3. Results

### 3.1. Protective Effects of CUR on UVB-Induced Tumorigenesis in SKH-1 Hairless Mouse

Exposure of mice to 180 mJ/cm^2^ UVB dose twice per week caused 100% tumor incidence at 20 weeks in UV alone group (Figure 1(a)); however, it took 23 weeks for post-CUR (UV + CUR-T)- and 25 weeks for pre-CUR (CUR-T + UV)-treatment groups. The first tumor appearance in UV-alone animals occurred at 16th week, which was delayed by 2 weeks in UV + CUR-T and 4 weeks in CUR-T + UV groups. No tumors were detected in un-irradiated control and CUR-T groups. Compared with UV alone group (Figure 1(b)), both CUR-T + UV and UV + CUR-T groups showed approximately 62% and 53% decrease in number of tumors per mouse throughout the experiment. Tumor volume per mouse per tumor (Figure 1(c)) was also decreased from 388 ± 44 mm^3^ in UV alone group to 126 ± 13 mm^3^ in CUR-T + UV and 152 ± 23 mm^3^ in UV + CUR-T groups, accounting for 68% and 59% decrease, respectively. None of the CUR treatments caused any significant decrease in diet consumption (data not shown) or body weight change (Figure 1(d)) compared with control mice. These results convincingly indicate the protective effect of topically applied CUR against UVB-induced tumorigenesis in SKH-1 mouse skin without any observable toxicity. 

### 3.2. CUR Inhibits UVB-Induced Apoptosis and Apoptotic Sunburn Cell Formation

**Figure 2:**
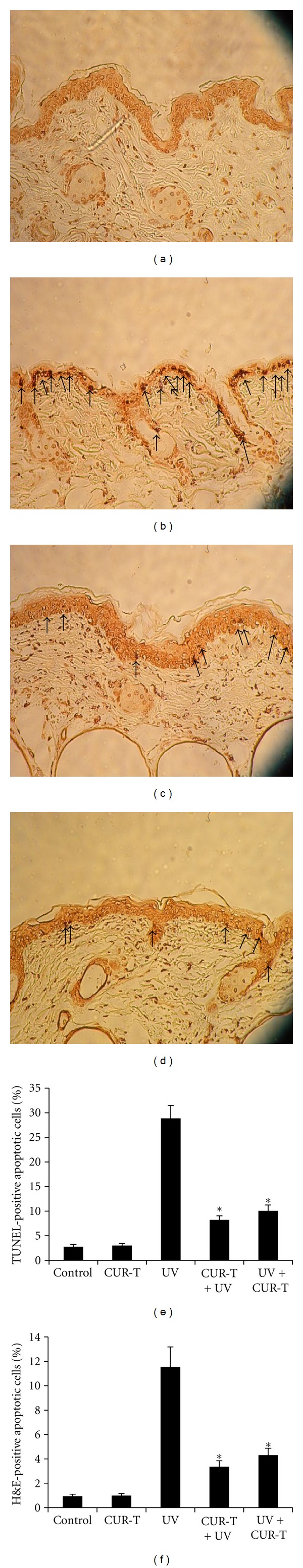
Inhibition of UVB-induced apoptosis by CUR. The apoptotic cells were detected by TUNEL assay and H&E staining (a) control; (b) UV; (c) CUR-T + UV; (d) UV + CUR-T. Arrows show apoptotic cells with brown staining. Quantitative results of TUNEL-positive cells (e), and H&E-positive cells (f) were statistically analyzed. Data were calculated as mean ± SE of 25 fields/5 mice/group. *P < 0.002 (TUNEL), *P < 0.005 (H&E).

Compared with unexposed control mice (Figure 2(a)), UVB exposure alone significantly increased TUNEL-positive apoptotic cells (b). Topical CUR treatment (CUR-T) by itself did not induce TUNEL-positive apoptotic cells *per se* (data not shown). However, both CUR-T + UV (c) and UV + CUR-T (d) showed a significant suppress in UVB-induced apoptosis. Consistent with the above results, quantitative analysis (e) revealed that UVB irradiation resulted in 28.85 ± 2.61% TUNEL-positive apoptotic cells, while low level of these apoptotic cells was observed in un-irradiated control (2.75 ± 0.49%) or CUR-T (3.14 ± 0.42%). Furthermore, both CUR-T + UV and UV + CUR-T resulted in 8.2 ± 0.85% and 9.7 ± 0.67% TUNEL-positive apoptotic cells accounting for 72% and 68% inhibition (e).

Characteristic dyskeratotic sunburn cells with pyknotic nuclei were detected by histomorphologic analysis using H&E staining. In parallel with TUNEL assay, H&E staining of apoptotic sunburn cells (Figure 2(f)) were markedly increased from approximately 0.94 ± 0.16% both in unexposed control and CUR-T alone to 11.55 ± 1.63% in UVB-exposed mice. Animals in CUR-T + UV and UV + CUR-T groups showed a significant reduction of sunburn cells to 3.36 ± 0.49% and 4.1 ± 0.32%, respectively, accounting for 71% and 65% inhibition. 

### 3.3. Protective Effects of CUR on UV-Induced Thymine Dimers

**Figure 3:**
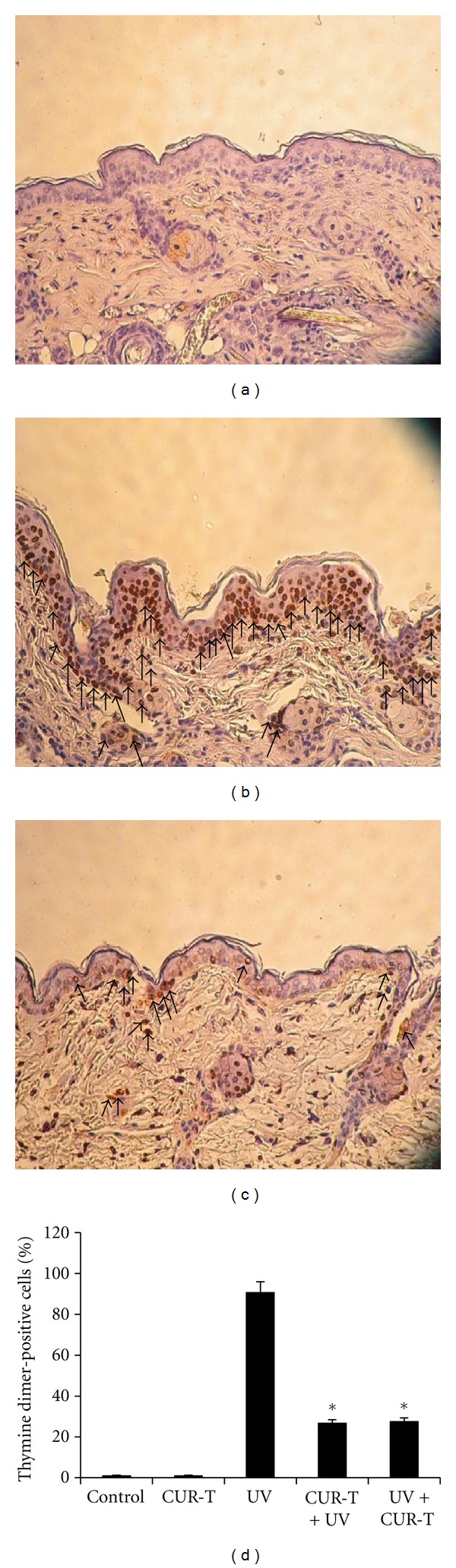
Inhibition of UVB-induced thymine dimer formation by CUR (a) control; (b) UV; (c) (CUR-T + UV). Arrows show thymine dimer-positive cells with brown staining. The results of UV + CUR-T treatment were similar to that of CUR-T + UV (data not shown). Thus, the data of UV + CUR-T in the subsequent figures were not shown but presented in the quantitative data. (d) is the quantitative results of thymine dimer-positive cell populations. *P < 0.001.

Thymine dimers are considered as an early and important biomarker for UVB-induced DNA damage. Previous study showed that UVB-induced thymine dimer formation in the epidermis peaks at 1 h after UVB exposure [21]. Compared with sham irradiated controls (Figure 3(a)), a single exposure of mice to UVB strongly induced the formation of thymine dimer-positive cells (b). In contrast, topical CUR application prior to UVB irradiation (CUR-T + UV) (c) resulted in a remarkable reduction in thymine dimer-positive population. More intense staining for thymine dimers was observed in the suprabasal layer than in the basal layer. Fewer stained cells and less intense staining for thymine dimers were observed in the dermis than in the epidermis. CUR-T by itself had no effect on biomarkers *per se*. Quantitative analysis (Figure 3(d)) showed that UVB irradiation resulted in 91 ± 4.95% thymine dimer-positive cells in epidermis, while negligible level of these cells were observed in unirradiated controls or CUR-T group. In contrast, CUR-T + UV and UV + CUR-T groups showed similar level of reduction (27.1 ± 1.41) in thymine dimer-positive cells (*P < 0.001). These results indicated that topical application of CUR prior to and immediately after UVB irradiation could protect the epidermis against UVB-induced damage, at least in part, through suppression of thymine dimer formation.

### 3.4. Effects of CUR on UVB-Induced Upregulation of p53-p21/Cip1 Cascade

Previous report indicated that in response to DNA damage by UV irradiation, p53 and p21/Cip1, are upregulated for cell cycle arrest to facilitate DNA repair [22, 23]. Peak increases in the number of p53-positive epidermal cells occurred at 8–12 h after exposure to UVB [22, 23]. Thus, skin samples taken at 8 h after UVB exposure were used for this assay. 

**Figure 4:**
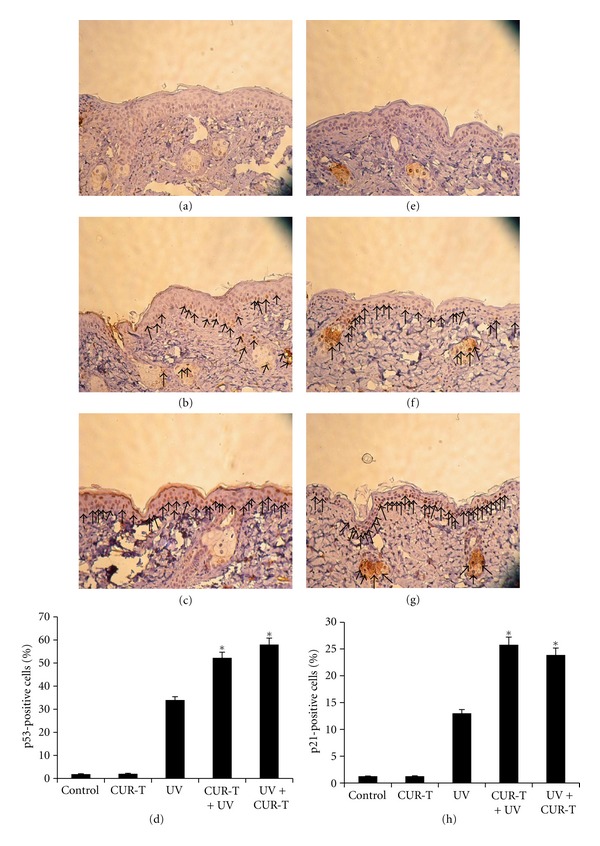
Effect of curcumin on UVB-induced p53 and p21/Cip1 expressions. CUR enhances UVB-induced p53 and p21/Cip1 expressions, left and right panels, respectively. (a) and (e) controls; (b) and (f) UV; (c) (g) CUR-T + UV. Arrows show p53-positive cells ((b) and (c)) or p21/Cip1-positive cells ((f) and (g)) with brown staining. (d) and (h) are the quantitative results of p53- and p21/Cip1-positive cells cell populations in five different experimental conditions, respectively. *P < 0.004 (p53), *P < 0.003 (p21/Cip1).

As shown in Figure 4, left panels, compared with unexposed and untreated control mice (a), UVB exposure alone remarkably increased the p53-positive cells (b), which were seen primarily in the basal layer, but some were also observed in the suprabasal layer of the epidermis near the basal layer. Topical application of CUR prior to UVB exposure further increased the numbers of p53-positive cells (c). As expected, topical CUR treatment alone did not affect p53-positive cells *per se* (data not shown). Quantitative analysis Figure 4(d), revealed that UVB irradiation resulted in 33.9 ± 1.41% p53-positive cells in epidermis, while low level of these cells were observed in unirradiated control (1.85 ± 0.21), or CUR-T group (2.04 ± 0.28%). Moreover, topical application of CUR prior to (CUR-T + UV) and immediately after (UV + CUR-T) UVB exposure resulted in 52.3 ± 2.47% and 57.8 ± 2.57% p53-positive cells, respectively, accounting for 54 and 70% enhancement (*P* < 0.004).

The same samples used for p53 detection were also analyzed for p21/Cip1-positive cells. Similarly shown in Figure 4, right panels, compared with unexposed control mice (e), UVB exposure alone significantly increased the p21/Cip-1-positive cells (f). Moreover, the CUR-T + UV group (g) further increased significantly in numbers of p21/Cip1-positive cells. Quantitative analysis (h) revealed that UVB irradiation resulted in 13.02 ± 0.71% p21/Cip1-positive cells in epidermis, which were markedly increased to 25.8 ± 1.41% in CUR-T + UV and 23.9 ± 1.27% in UV + -CUR-T group, accounting for 98 and 84% enhancement (*P* < 0.003). 

### 3.5. CUR Suppresses UV-Induced Cell Proliferation

**Figure 5:**
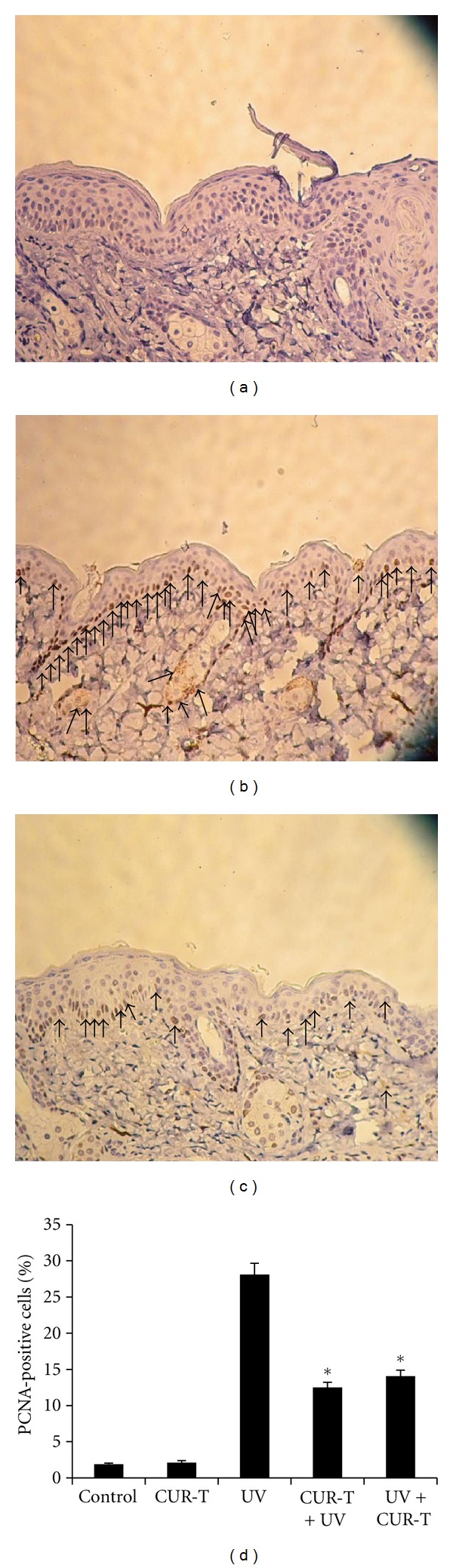
Inhibition of UVB-induced PCNA-positive cells by CUR (a) control; (b) UV; (c) CUR-T + UV. (d) is the quantitative results of PCNA-positive cell populations in five different experimental conditions. *P < 0.002.

We next examined the effect of UVB exposure without or with CUR treatments on proliferation status of epidermis by measuring PCNA level. Figure 5 showed that compared with unexposed control mice (a), UVB exposure alone significantly increased the PCNA-positive cells (b). However, significantly decreased the numbers of PCNA-positive cells was observed in CUR-T + UV group (c). Compared with low levels of PCNA-positive cells observed in unirradiated controls (1.9 ± 0.1%) and CUR-T mice (2.1 ± 0.28%), UVB irradiation resulted in 28.1 ± 1.56% PCNA-positive cells in epidermis (Figure 5(d)), which were reduced to 12.5 ± 0.71 in CUR-T + UV and 14.9 ± 0.8% in UV + CUR-T groups, accounting for 56 and 51% inhibition (*P* < 0.002).

### 3.6. CUR Inhibits UVB-Induced Activation of NF-*κ*B, COX-2, NO, and PGE2 Levels

**Figure 6:**
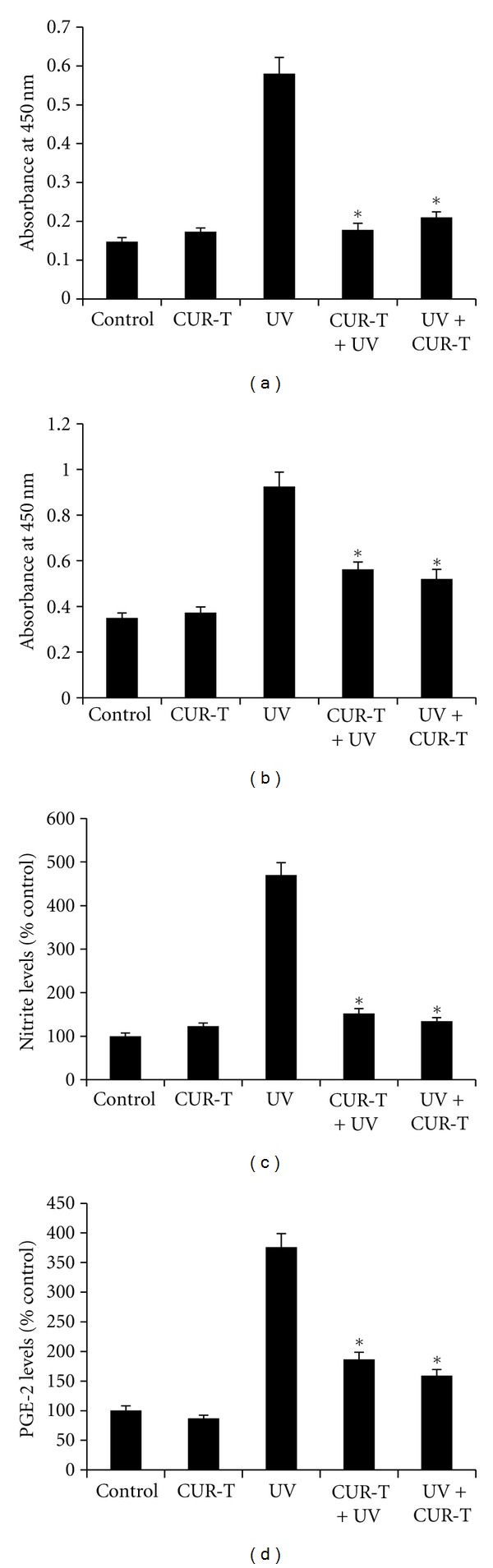
Inhibition of UVB-induced activation of NF-*κ*B, COX-2, NO, and PGE2 expressions by CUR. Skin lysates were analyzed for NF-*κ*B, COX-2, NO, and PGE2 activities (a) NF-*κ*B; (b) COX-2; (c) NO; and (d) PGE2. Activities were calculated as mean ± SE (*n* = 5). *P < 0.002 (NF-*κ*B), *P < 0.005 (COX-2), *P < 0.001 (NO), *P < 0.002 (PGE2).

We then examined effects of UVB exposure without or with CUR treatments on cellular factors associated with skin tumorigenesis.  Figure 6(a) showed that UVB exposure alone significantly increased the activity of NF-*κ*B (0.58 ± 0.04), compared with unexposed control mice (0.15 ± 0.01) or CUR-T mice (0.17 ± 0.01). However, NF-*κ*B activity in CUR-T + UV and UV + CUR-T groups were significantly decreased to the control levels.

Figure 6(b) showed an increased COX-2 activity (0.93 ± 0.06) in response to UVB irradiation, but was significantly decreased in CUR-T + UV (0.56 ± 0.0) and UV + CUR-T (0.52 ± 0.02). Similar background levels of COX-2 activity were observed in unirradiated control (0.35 ± 0.02), or CUR-T group (0.37 ± 0.03). 

UVB exposure alone resulted in NO activity of 470 ± 28.3% relative to that in the un-irradiated control (102 ± 12) and CUR-T (109 ± 13), accounting for approximately 4.6-fold increase (Figure 6(c)). However, the activity was decreased to the control level in CUR-T + UV and UV + CUR-T groups.  Figure 6(d) showed that PGE2 activity (375 ± 14) was marked increase above the control levels in response to UVB irradiation, but was significantly decreased by topical application of CUR prior to (175 ± 4) and immediately after (150 ± 3) UVB irradiation, resulting in net reduction of 53 and 60% of PGE2 activity, respectively. Topical CUR treatment by itself did not affect the activity of PGE2 *per se*.

## 4. Discussion

The major findings in the present study are that CUR caused delay and reduction in UVB-induced tumor appearance, multiplicity, and size in hairless mice without any toxicity. The photoprotective effect of CUR could occur at several mechanistically different levels. Our results further showed that CUR protects SKH-1 hairless mice skin from UVB-induced DNA damage and that UVB-caused cell proliferation and apoptotic sunburn cell formation are prevented by CUR possibly via further induction in p53-p21/Cip1 cascade. It is anticipated that the antiproliferative effect of CUR against UVB-induced tumor might also involve cell cycle regulatory mechanisms. Our results showed that CUR increased the protein expression of Cdk inhibitor, Cip1/p21, which is well known to interact with and inhibit kinase activity of Cdk-cyclin complex. In our study, CUR treatment resulted in a further increase in UV-induced p53 accumulation with a concomitant increase in p21/Cip1 protein levels, which is in accord with the inhibition of UV-induced cell proliferation and apoptosis by CUR, suggesting their possible role in cell growth inhibition rather than apoptosis induction.

It is well known that sunburn cells are formed in the mammalian epidermis after exposure to UV radiation. These cells have distinct morphology as having a shrunken, homogenized, densely staining cytoplasm, and a hyperchromatic condensed pyknotic nucleus. As demonstrated in this study, these features were readily seen with routine H&E staining using light microscopy. It has been demonstrated that sunburn cells are apoptotic cells and contain a hall-mark of apoptosis, namely, DNA strand breaks observed by end-labeling. 

Reactive oxygen species (ROS) and other free radicals are able to interact with DNA to induce mutations and DNA-base modifications. This oxidative damage represents the initial step of carcinogenesis when cellular repair mechanisms fail to fix these lesions and result in either modulation of gene expression through epigenetic effects or in permanent somatic mutations and chromosomal rearrangements. One of the most important characteristics of UV-caused carcinogenesis is DNA damage and mutagenesis, and thymine dimers are known as “hot spots” of UV mutagenesis [1]. Our study showed CUR treatment remarkably decreased in UV-induced thymine dimer-positive cells. CUR treatment may result in increase in mismatch repair enzyme MSH2. Previous report showed that DNA mismatch repair system is inactivated by oxidative stress [24]. The antioxidant property of CUR [8, 9] suggests that suppression of oxidative stress by CUR could be one of the possible mechanisms that resulted in the activation of repair enzymes much earlier as compared with UV alone; thereby it is possible that thymine dimers were removed much earlier in CUR-treated animals than 1 h, the time point used in our study. More studies are needed in future as a function of time employing different time points to assess whether CUR causes a faster repair of UV-induced DNA damage ultimately leading to a strong reduction in thymine dimer-positive cells. The other possibilities could be that CUR protects epidermal cells from UV-induced thymine dimer-positive cells by modulating DNA repair enzymes other than MSH2 and/or by an alteration in ATM/ATR pathways. It is not known whether CUR effect on these pathways as an upstream response for its efficacy against UV-induced thymine dimer-positive cells in epidermis. 

p53 plays an important role in growth arrest and apoptosis. In response to DNA damage by UV irradiation, p53 is upregulated to arrest cell cycle through transcriptional activation of p21/Cip1 to facilitate DNA repair when the damage is mild, or induce apoptosis via activating apoptotic proteins (such as Fas/Apo-1, Bax, and DR5), or downregulating antiapoptotic proteins (such as cellular inhibitor of apoptosis protein 2 and bcl-2) when the damage is severe [25, 26]. In the present study, we showed that CUR upregulated p53 with a concomitant increase in p21/Cip1 protein levels and decrease in apoptotic sunburn cells of UVB-irradiated skin. In addition, we further observed that UVB-induced PCNA-positive cells were inhibited by CUR treatment. These findings establish a relationship between the formation of sunburn cells and apoptosis-related biomarkers. These results are in accord with the notion that inhibition of cell proliferation could be one of the mechanisms by which CUR protects damaged cells from entering the cell cycle, so that damaged cells have sufficient time for repairing in case the damage is severe. Collectively, the proposed mechanisms for the protective effect of CUR on UV-induced photodamage in epidermal cells are as following. CUR protects SKH-1 mouse epidermis from DNA damaging effect of UV such as thymine dimer-positive cells, thereby decreasing UV-caused apoptotic/sunburn cells. Further, CUR inhibits UV-induced epidermal cell proliferation via decreasing PCNA possibly through activation of p53-p21/Cip1, suggesting a cell growth delay rather than acceleration of cell death. 

UV irradiation also results in the formation of ROS and prostaglandins [27, 28]. UV-induced prostaglandins may play important roles in inflammation, photoaging, and photocarcinogenesis in human skin. NO and prostaglandins, which are produced by iNOS and COX-2, respectively, have been implicated as important mediators in the processes of inflammation [29]. Thus, potential inhibitors of iNOS and COX-2 have been considered effective therapeutically for preventing inflammatory reaction and disease. Our results in this study clearly demonstrated that CUR significantly suppressed UV-induced NO, COX-2, and PGE2 levels in the mouse skin. Control of COX-2 induction involves a complex array of regulatory factors including NF-*κ*B [29]. We found that topical application of CUR was effective in terms of inhibiting NF-*κ*B DNA binding. The inhibitory effect of CUR on NF-*κ*B activation by UVB could be due to inhibition of I*κ*B degradation and p65 translocation to the nucleus [20]. 

In this study, we used a single large dose (180 mJ/cm^2^) of UVB given to SKH-1 hairless mice, which is approximately six times higher UVB dose than the physiologically relevant dose (30 mJ/cm^2^/day of UVB). One reason for selecting a single large dose of UVB is logical, including less stress on the animals. Nonetheless, we believe that a single large dose is well justified scientifically for outdoor occupational exposure of humans to UVB or a sunbathing exposure in the summer was reported to range from 50–100 mJ/cm^2^ per day [30]. We hypothesized that if CUR treatment can protect animals from UVB radiation after a single large dose of exposure, it is reasonable to expect that it could protect against fractionated, protracted irradiation. Studies involving the simulation of solar optical radiation on a laboratory scale have taken advantage of the characteristics of xenon arc emission. Since the dose of UVR used in the standardized model is sufficient to cause tumors in all mice, the results of this study justify UVB as the main carcinogen for the tumors observed in this study.

In conclusion, our data clearly indicate that topical application of CUR inhibits UVB-induced carcinogenesis and decreases several UVB-induced biomarkers. In the present study, we observed that application of CUR both prior to and immediately after UVB irradiation showed equally or a moderately better protective effect against some of the UVB-caused alterations in SKH-1 mouse epidermis. These results indicated that CUR can inhibit UVB-induced carcinogenesis by mechanisms independent from its possible “sun-screening” effects.

The molecular events associated with protective effect of CUR in UVB-induced skin cancer including downregulation of cell proliferative controls, involving thymine dimer, PCNA, apoptosis, transcription factors NF-*κ*B, and inflammatory responses involving COX-2, PGE2, and NO, while upregulation of p53 and p21/Cip1 to facilitate DNA repair. CUR efficacy observed in the present study in terms of a decrease in tumor number and shrinkage of tumor size would have potential clinical significance. 

## 5. Conclusion

Collectively, the present study provides fundamental information on the effects of CUR on mechanistically important early biomarkers for UVB-caused effects *in vivo*, suggesting a short-term model for evaluation of potential protective pharmacological modulators against UVB-induced damages. Our results provide a focus for the rational development of CUR as a safe and effective chemopreventive agent against UV-induced photoaging and photocarcinogenesis.
